# Multishelled NiO Hollow Microspheres for High-performance Supercapacitors with Ultrahigh Energy Density and Robust Cycle Life

**DOI:** 10.1038/srep33241

**Published:** 2016-09-12

**Authors:** Xinhong Qi, Wenji Zheng, Xiangcun Li, Gaohong He

**Affiliations:** 1 State Key Laboratory of Fine Chemicals, Chemical Engineering Department, Dalian University of Technology Linggong Road 2#, Dalian 116024, China

## Abstract

Multishelled NiO hollow microspheres for high-performance supercapacitors have been prepared and the formation mechanism has been investigated. By using resin microspheres to absorb Ni^2+^ and subsequent proper calcinations, the shell numbers, shell spacing and exterior shell structure were facilely controlled via varying synthetic parameters. Particularly, the exterior shell structure that accurately associated with the ion transfer is finely controlled by forming a single shell or closed exterior double-shells. Among multishelled NiO hollow microspheres, the triple-shelled NiO with an outer single-shelled microspheres show a remarkable capacity of 1280 F g^−1^ at 1 A g^−1^, and still keep a high value of 704 F g^−1^ even at 20 A g^−1^. The outstanding performances are attributed to its fast ion/electron transfer, high specific surface area and large shell space. The specific capacitance gradually increases to 108% of its initial value after 2500 cycles, demonstrating its high stability. Importantly, the 3S-NiO-HMS//RGO@Fe_3_O_4_ asymmetric supercapacitor shows an ultrahigh energy density of 51.0 Wh kg^−1^ at a power density of 800 W kg^−1^, and 78.8% capacitance retention after 10,000 cycles. Furthermore, multishelled NiO can be transferred into multishelled Ni microspheres with high-efficient H_2_ generation rate of 598.5 mL H_2_ min^−1^ g^−1^_Ni_ for catalytic hydrolysis of NH_3_BH_3_ (AB).

Multishelled metal oxide hollow microspheres with high surface area, low ion transfer resistance, and enhanced energy storage capacity have attracted considerable attentions as safe, inexpensive anode materials for supercapacitors^1^,^2^,^3^,^4^. NiO has been considered to be one of the most attractive electrode materials for supercapacitors due to its low cost, high availability and high theoretical specific capacitance (3750 F g^−1^)^2^. Thus, various NiO nanostructures such as hollow microspheres^5^, nanoparticles^6^, nanosheets^7^, nanotube arrays^8^, have been successfully fabricated for energy storage. However, most of the available electrode materials possess relatively simple configurations and complex synthesis procedures. In addition, comparatively low capacitance (<1000 F g^−1^) and energy density (<50 Wh kg^−1^) has been usually achieved previously owing to their low accessible surface areas^5^,^7^,^9^. Multishelled hollow microspheres with designed shell number, shell spacing, and exterior shell structure are ideal candidates for high performance anode materials^3^,^10^: (1) the unique hollow structure with high specific surface area provides more accessible faradic reactive sites in real capacitive process, leading to higher energy density; (2) the shells with porous structure and large empty space between neighboring shells facilitate electrolyte penetration and accessibility of the electrolyte to the internal active surface, shorten electron/ion diffusion length and thus high rate capability and power density are obtained; (3) different shells are supported by each other and the exterior shell protects the interior shells from the electrochemical dissolution, leading to structural and electrochemical stabilities of the electrodes and thus cycling performance is improved^1^,^3^,^4^,^10^,^11^,^12^. Consequently, researchers are currently more focused on the design and fabrication of hollow structures with higher structure complexity, hoping to achieve optimized physical/chemical properties for specific applications. To date, however, as one of the most promising energy storage materials, multishelled NiO hollow spheres with controlled shell spacing, exterior shell structure, and high energy storage ability have not been reported, due in part to the difficulties in controlling shell numbers and shell spacial arrangements.

Herein, for the first time, we report a facile approach to fabricate multishelled NiO hollow microspheres for high-performance supercapacitors. By using resin microspheres to absorb Ni^2+^ ions and subsequent proper calcination, single-, double-, triple-, and quadruple-shelled NiO hollow microspheres were prepared via varying synthetic parameters such as Ni^2+^ ion concentration and calcination rate. Particularly, compared to the previous reported metal oxide hollow spheres^1^,^3^,^4^,^10^,^11^,^12^, the exterior shell structure that accurately associated with the ion transfer was finely controlled by forming a thin single shell or closed exterior double-shells. The unique NiO hollow microspheres with controlled internal multishells, shell spacing, and exterior shell structure have made a significant contribution to ion transfer in hollow spheres and to construction of high-performance energy storage devices. In addition, the solvent infusion and subsequent calcination approach here are obviously easier than the previous hydrothermal or layer-by-layer coating method in controlling the structural parameters^1^,^2^,^6^,^10^,^12^,^13^. Thus for the preparation of multishelled hollow spheres, a simple and novel fabrication approach is obtained. Notably, the triple-shelled NiO hollow microspheres with an outer single-shelled structure show a remarkable capacity of 1280 F g^−1^ at 1 A/g, ultra-high energy density of 51.0 Wh kg^−1^ at a power density of 800 W kg^−1^ (based on total mass of the active materials from both electrode materials), and long recycling stability (78.8% capacitance retention after 10,000 cycles for NiO//RGO@Fe_3_O_4_ aqueous asymmetry supercapacitors). Even at a high power density of 8,000 W kg^−1^, the capacitor still delivers an energy density of 22.7 Wh kg^−1^. In addition, the multishelled NiO can be transferred into multishelled Ni hollow microspheres with a high-efficient H_2_ generation rate of 598.5 mL H_2_ min^−1^ g^−1^_Ni_ from catalytic hydrolysis of NH_3_BH_3_ (AB) aqueous solution.

## Results and Discussion

**Figure 1:**
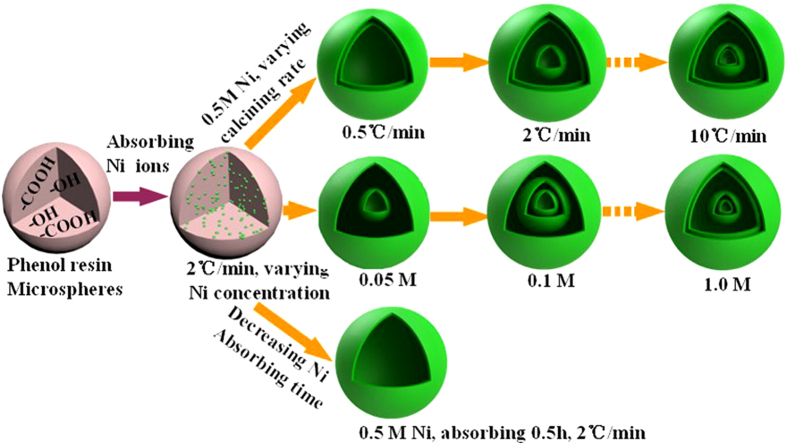
Preparation of various multishelled NiO hollow microspheres by controlling the calcination rate, Ni precursor concentration and absorption time of Ni ions.

A mild hydrothermal method is developed to prepare uniform resin microspheres (RF) using 2,4-dihydroxybenzoic acid as the precursor in Supplementary Fig. S1. Due to the presence of numerous –COOH and OH functional groups in the precursor molecules, the Ni^2+^ ion penetration and combination with the resin microspheres should be facilitated effectively^14^. The synthesis of multishelled NiO spheres is schematically illustrated in Fig. 1. Single-, double-, triple-, and quadruple-shelled NiO hollow microspheres with controlled exterior shell structure can be facilely obtained by adjusting the synthetic parameters such as Ni ion concentration, ion absorbing time and calcination rate of the Ni^2+^ ion infused resin microspheres. The formation mechanism will be discussed as follows.

**Figure 2:**
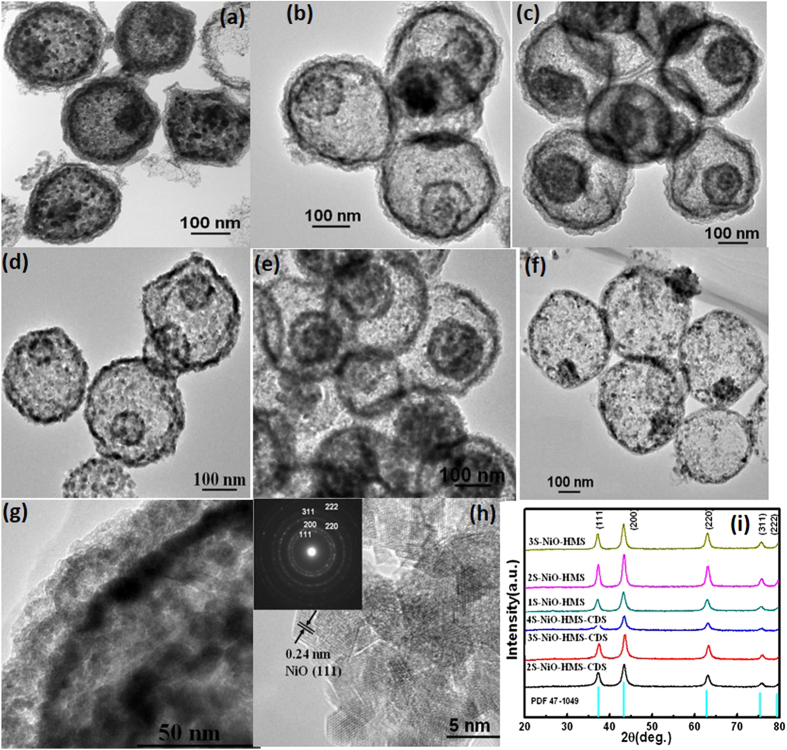
TEM images of (**a**–**c**) double-, triple-, and quadruple-shelled hollow NiO microspheres with closed exterior double-shells (0.5 M Ni^2+^, 2S-, 3S-, and 4S-NiO-HMS-CDS at 0.5, 2, and 5 °C/min); (**d**–**f**) double-, triple-, and single-shelled NiO hollow microspheres; (2S-, 3S, and 1S-NiO-HMS at 0.05 M, 0.1 M, and 0.5 M, 0.5 h, 2 °C/min); (**g**,**h**) HRTEM images of the closed exterior double-shells and NiO crystal structure; (**i**) XRD patterns of various NiO hollow spheres.

Transmission electron microscopy (TEM) images (Fig. 2a–c) confirm that double-, triple-, and quadruple-shelled NiO hollow microspheres with closed exterior double-shells were synthesized (2S-, 3S-, and 4S-NiO-HMS-CDS) by soaking RF templates in a 0.5 M Ni(NO_3_)_2_ solution with subsequent proper annealing (0.5, 2, or 5 °C min^−1^ to 550 °C and held for 4 h). And the quadruple-shelled NiO hollow microspheres were also obtained at the heating rate even up to 10 °C min^−1^ (Supplementary Fig. S2a). With the increase of heating rate, the temperature gradient along the radial direction of the RF microspheres is increased, resulting in two opposite directional forces existing between the outer NiO shell and the inner penetrated RF. One is the adhesion force in outward direction and the other is the contraction force by decomposition of the inner core. The two forces contribute to the formation and separation of the core and shell. The fast heating rate may increase the layer-by-layer removal rate of the resin microsphere templates, which favors the separation between adjacent NiO layers, leading to the formation of multi-layers. In addition, double and triple-shelled NiO hollow microspheres with large shell spacing (2S-, 3S-NiO-HMS, Fig. 2d,e) were obtained upon soaking RF microspheres in 0.05 M and 0.1 M Ni^2+^ solution respectively (2 °C min^−1^). The low concentration of Ni^2+^ leads to less Ni^2+^ on the surface of RF, contributing to the formation of exterior single shell. The structure variation demonstrates that the Ni^2+^ ions concentration plays a dominant role on the shell spacing. When high Ni^2+^ concentration is used for the immersion of resin template surface, multishelled NiO hollow spheres with closed exterior double-shells are obtained. This can be further confirmed by increasing the Ni^2+^ concentration to 1.0 M (Supplementary Fig. S2b), followed by the same annealing process (2 °C min^−1^ to 550 °C and held for 4 h). Furthermore, only single-shelled NiO hollow spheres was synthesized (Fig. 2f) with decreasing the soaking time from 7 h to 0.5 h in a 0.5 M Ni^2+^ solution, which could be attributed to the low immersion Ni^2+^ concentration and short soaking time. TEM image in Fig. 2g reveals that the shells have a hierarchical porous structure and are composed of interconnected NiO grains. The diameters of these grains are approximately 20–30 nm, which are determined by TEM and Scherrer analysis of peak broadening. The XRD patterns for all hollow microspheres (Fig. 2i) clearly show the same reflections with pure phase of cubic NiO (PDF #47-1049). This was further confirmed by HRTEM image (Fig. 2h), which demonstrates that the nanocrystal subunits have the lattice spacing consistent with NiO^15^. The selected-area electron diffraction (SAED) pattern displays well-defined diffraction rings, suggesting the polycrystalline characteristics of NiO. The SAED results are well in agreement with the HRTEM image and XRD pattern.

**Figure 3:**
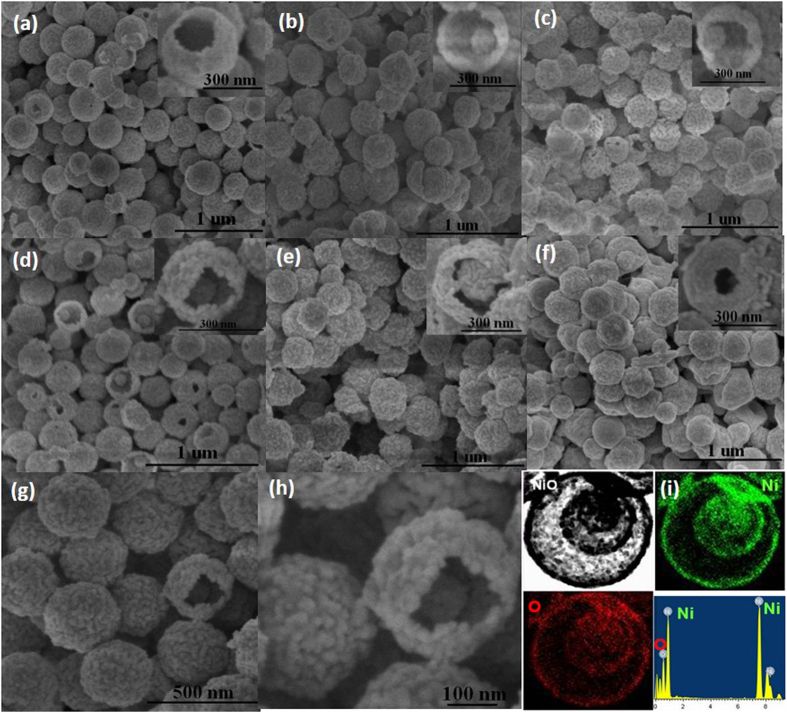
(**a–c**) SEM images of the 2S-, 3S-, and 4S-NiO-HMS-CDS; (**d–f**) 2S-, 3S-, and 1S-NiO-HMS; (**g**,**h**) HRSEM images of the 4S-NiO-HMS-CDS, (**i**) energy dispersive X-ray spectroscopy (EDS) elemental mapping of 3S-NiO-HMS.

Field-emission scanning electron microscopy (FESEM) images in Fig. 3 show that all the NiO hollow microspheres have a uniform size of ~350 nm. The ball-in-ball multishelled structure is clearly confirmed from the broken spheres. Unfortunately, the closed exterior double-shells cannot be easily distinguished from the FESEM images due to their closed space. The HRSEM images (Fig. 3h) show that the shells are composed of NiO nanocrystal grains of ~25 nm in diameter, in consistence with the TEM results (4S-NiO-HMS-CDS). The energy dispersive X-ray spectroscopy (EDS) elemental mapping (Fig. 3i) also suggests the formation of triple-shelled NiO hollow spheres with a high purity (3S-NiO-HMS). To illustrate the formation mechanism of these novel metal oxide nanostructures, TEM, scanning transmission electron microscope (STEM) and EDS elemental mapping and line scanning were used to monitor the morphological evolution and formation process as a function of the annealing time. It can be seen (Supplementary Fig. S3a–c) that the closed exterior double-shells are firstly formed after calcining the Ni^2+^ infused resin microspheres for 0.5 h (soaking in a 0.5 M Ni^2+^ solution for 7 h, annealing at 5 °C/min, and held at 550 °C for 0.5 h), and the interior part is composed of Ni oxides-carbon composites from the mapping and line-scanning. The color change of the obtained product from initial brown to black also demonstrates the formation of NiO on the template surface. After heat treatment at 550 °C for 1.5 h (Supplementary Fig. S3d–f), the shell space can be clearly seen from the TEM images with further decomposition of the resin template. When prolonging the calcination duration to 3 h, quadruple-shelled NiO hollow microspheres are obtained accompanying the almost complete removal of the templates (the carbon content in the composites decreases from 36.94 wt% to 2.1 wt% with increasing heating time from 0.5 h to 3 h). Thus, to get pure NiO crystal, the templates were held at 550 °C for 4 h in this work. The morphological evolution study confirms that the shells are formed layer by layer from the outer surface of the templates to the interior with the gradual decomposition of the templates, during which primary NiO nanoparticles are interconnected together to form porous shells.

The specific surface area and the corresponding Barrett-Joyner-Halenda (BJH) pore size distributions of the hollow microspheres were investigated by using N_2_ adsorption/desorption analysis (Supplementary Fig. S4). All the isotherm profiles can be categorized to type IV with a small hysteresis loop^16^,^17^. The BJH pore size distribution curves indicate that there are abundant mesopores (3.3–4.3 nm) existing in these NiO samples (likely formed between nanoparticles). The presence of these mesopores may facilitate electrolyte transport, OH^-^ ions diffusion and strain release.

**Figure 4:**
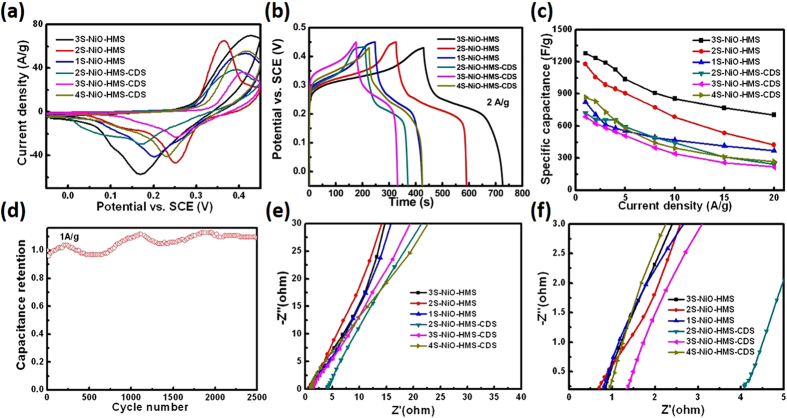
(**a**,**b**) Cyclic voltammetry (50 mV s^−1^) and galvanostatic charge/discharge properties of the multishelled NiO hollow microspheres, (**c**) specific capacitance of the hollow microspheres material under different current densities, (**d**) cycling stability of the 3S-NiO-HMS electrode materials, (**e**,**f**) EIS plots of the NiO hollow microsphere electrode materials.

The capacitive properties of NiO hollow microspheres are evaluated by using a three-electrode system in 2.0 M KOH solution, and the results are shown in Fig. 4 and Supplementary Fig. S5. Figure 4a gives the cyclic voltammetry (CV) curves of the hollow spheres at scan rate of 50 mV s^−1^ (−0.05–0.45 V), showing that the capacitance of the materials mainly results from the faradic capacitance caused by the fast and reversible redox reaction of NiO (NiO + OH^−1^ = NiOOH + e^−1^)^4^,^18^. For all hollow spheres (Supplementary Fig. S5), the current responses increase with the scan rate, indicating excellent capacitive behavior of the microspheres, which is ascribed to the facile ion diffusion and large specific surface area of the electrode materials^19^,^20^. The specific capacitances are 1236, 1057, 707, and 832, 622, 660 F g^−1^ at 2 A g^−1^ for the 3S-, 2S-, 1S-NiO-HMS, and 4S-, 3S-, 2S-NiO-HMS-CDS respectively from the discharge curves in Fig. 4b (−0.05–0.45 V, the potential window is −0.05–0.43 V for samples 2S- and 4S-NiO-HMS-CDS due to the oxygen evolution reaction). The results are consistent with the CV curves in Fig. 4a, which implies that the higher capacitance of electrode materials has larger areas enveloped by the CV curves. The specific capacitances derived from the discharge curves at different current densities are summarized in Fig. 4c. It can be seen that 3S-NiO-HMS has a highest capacitance of 1280 F g^−1^ at 1 A g^−1^, and still keeps a high value of 704 F g^−1^ at 20 A g^−1^, indicating good rate capacity of the NiO electrode materials. Moreover, the capacitances of 1178 and 1280 F g^−1^ at 1 A g^−1^ for the 2S- and 3S-NiO-HMS reported here are much higher than those of the double- and triple-shelled NiO hollow microspheres (555.4 and 381.7 F g^−1^ at 1 A g^−1^) synthesized by a layer-by-layer self-assembly method and other NiO based electrode materials^2^,^6^,^7^,^8^,^9^. This can be ascribed to their high specific surface areas and pore volume (88 and 93 m^2^/g, Supplementary Table S1), thin exterior porous shell as well as unique multishelled structure. To the best of our knowledge, the capacitance of 1280 F g^−1^ for 3S-NiO-HMS supercapacitor at 1 A g^−1^ presented here is the best among pure NiO anode materials to date. The results confirm that the shell structures of the NiO hollow microspheres plays an important role in optimizing the capacitive performance. The proper shell structures provide more active sites and appropriate pore volume, both of which could facilitate the ion penetration and the process of redox reactions^3^. Cycle stability was measured by repeating charge/discharge process of the electrode materials (3S-NiO-HMS) at a current density of 1 A g^−1^ (Fig. 4d). The specific capacitance gradually increases, which is ascribed to activating process of the inner shells during the charge/discharge process^21^. And it reaches 108% of its initial value after 2500 cycles, demonstrating high stability of the NiO materials.

Nyquist plots of the multishelled NiO hollow microspheres were measured in an open-circuit condition (Fig. 4e,f). At high-frequency, the point intersecting with the real axis exhibits an intrinsic Ohmic resistance or equivalent series resistance (ESR) of the electrode materials^22^,^23^,^24^. From the inset figure, the internal resistances of 3S-, 2S-, 1S-NiO-HMS are 0.76 Ω, 0.60 Ω and 0.73 Ω, which are smaller than those of 0.86 Ω, 1.28 Ω and 4.08 Ω for 4S-, 3S-, and 2S-NiO-HMS-CDS respectively (for 4S-, 3S-, 2S-NiO-HMS-CDS, the whole resistance gets smaller when the number of shells increases because each shell forms parallel resistance)^1^,^25^. The results indicate that the hollow spheres with an exterior single shell and large shell spacing (3S-, 2S-, 1S-NiO-HMS) can decrease the intrinsic resistance of the electrodes and improve the electron transportation from active materials to the current collector. At low frequency, the linear line delivers a Warburg impedance involved in the diffusion of electrolyte ions. Evidently, the large slope of 3S-, 2S-, 1S-NiO-HMS suggests faster ion transport and more ideal capacitive behavior. While for 4S-, 3S-, 2S-NiO-HMS-CDS, the low slope reflects the high diffusive resistance of the electrolyte ions through the closed exterior double-shells, which should be responsible for their low capacitance performance, though these electrode materials have relatively high specific surface areas (94.2–117.8 m^2^/g, Supplementary Table S1). For 3S- and 2S-NiO-HMS electrode materials, their large shell spacing and exterior single-shell structure, fast ion/electron transfer, high specific surface area and pore volume (93.28 and 88.18 m^2^/g, 0.334 and 0.30 cm^3^/g, Supplementary Table S1) could guarantee an abundance of the active sites and the easy electrolyte penetration into the materials, resulting in improved capacitive performance^1^,^10^. Additionally, the multishelled hollow structure with hierarchical pores and the large shell spacing between neighboring shells facilitate the ions from both outer and inner shell to be charged and transferred, ensuring high utilization rate of the active material and high rate capability^11^.

**Figure 5:**
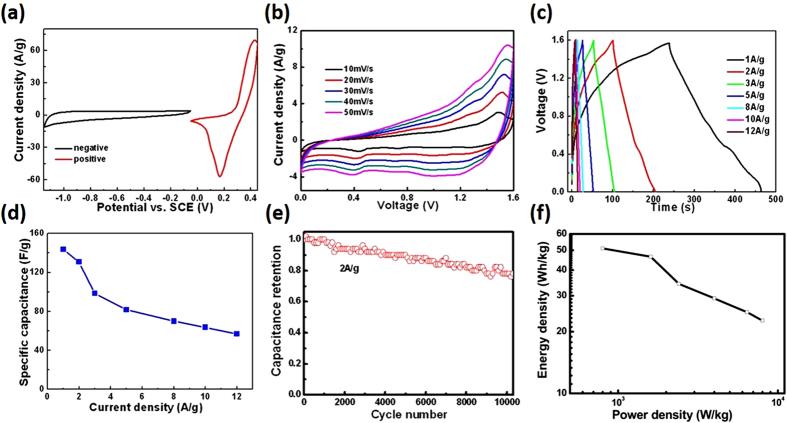
(**a**) CV curves collected for 3S-NiO-HMS hollow spheres and RGO@Fe_3_O_4_ electrodes at a scan rate of 50 mV/s, (**b**,**c**) CV curves of the 3S-NiO-HMS//RGO@Fe_3_O_4_ asymmetric capacitor at different scan rate, and the charge/discharge curves of the supercapacitor at different current densities, (**d**) the calculated specific capacitance, (**e**) cycling performance of the asymmetry capacitor at a current density of 2.0 A g^−1^, (**f**) Ragone plot of the 3S-NiO-HMS //RGO@Fe_3_O_4_ asymmetry capacitor.

To further evaluate the practical application performance of the multishelled NiO hollow microspheres, an asymmetric supercapacitor (ASC) device is fabricated. In this device, the 3S-NiO-HMS acts as the positive electrode, RGO@Fe_3_O_4_ nanocomposites (reduced graphene oxide and Fe_3_O_4_ composites) act as the negative electrode, and the electrolyte and separator are 2.0 M KOH aqueous solution and a porous polypropylene membrane, respectively. Fe_3_O_4_ was considered to be an ideal negative electrode material in the ASC due to its large theoretical capacity (~1000 mAh g^−1^) and low cost. However, the pure Fe_3_O_4_-based electrodes generally suffer from low rate capability and low capacitance of 60–80 F g^−1^. To optimize the performance of the negative electrode, RGO was selected as the matrix to anchor Fe_3_O_4_ nanoparticles, which can increase the electrical conductivity, prevent aggregations, and in turn ensure structural stability of the electrode^26^. The structures and electrochemical properties of the RGO@Fe_3_O_4_ nanocomposites were also evaluated (Supplementary Fig. S6). The typical CV curves of the NiO//RGO@Fe_3_O_4_ device at various scan rates (0–1.6 V) clearly show capacitance from both electric double-layer capacitance and pseudocapacitance (Fig. 5b)^4^. All the curves exhibit a similar shape in the scan rates of 10–50 mV/s, indicating excellently fast charge/discharge properties of the device. The stable galvanostatic charge/discharge curves were tested at cell voltage as high as 1.6 V (Fig. 5c) and the specific capacitance was calculated based on the total mass of active materials from both negative and positive electrodes (Fig. 5d). In addition, the asymmetric cell shows outstanding cycle properties at a current density of 2.0 A g^−1^ (Fig. 5e). Remarkably, the asymmetric supercapacitor manifests very high cycling stability and still delivers 78.8% of its initial capacitance even after 10,000 cycles. These results show that the high capacitive performance of the triple-shelled NiO electrode could guarantee the high capacitance and stability in the full-cell configuration. Long cycling life is an important requirement for supercapacitors. In this regards, NiO often suffers from a limited long-term stability because of its partial dissolution in the electrolyte during cycling, which restricts the commercial application of such low-cost materials as supercapacitor electrode. However, the construction of multishelled NiO hollow microspheres is able to noticeably meliorate the cycling performance. In the multi-shell structure of NiO hollow microspheres, the shells support for each other, and especially the exterior shell can protect the interior shells. This specific structure can undertake some mechanical deformation, decrease the swell in the redox processes and suppress the dissolution of NiO in electrolyte^1^,^3^,^10^.

Figure 5f shows a Ragone plot of the aqueous asymmetric supercapacitor assembled by the two-electrode full cell configuration. And the supercapacitor reveals an ultrahigh energy density of 51.0 Wh kg^−1^ at a power density of 800 W kg^−1^ (based on the total mass of active materials from the two electrodes). Even at a high power density of 8,000 W kg^−1^, the capacitor still delivers an energy density of 22.7 Wh kg^−1^. This excellent performance are superior to the most reported values of Ni, Co and Mn-based supercapacitors, as listed in Supplementary Table S2. Such as CNT/NiO//porous carbon polyhedrons (23.4 Wh kg^−1^ at 1,000 W kg^−1^)^7^, NiO//carbon (11.3 Wh kg^−1^ at 920 W kg^−1^)^25^, NiO-Ni//activated carbon (19.1 Wh kg^−1^ at 1,100 W kg^−1^)^9^, Ni(OH)_2_/graphene//graphene (30.0 Wh kg^−1^ at 800 W kg^−1^)^27^, NiCo_2_O_4_/Co_0.33_Ni_0.67_(OH)_2_//CMK-3-ASC (31.2 Wh kg^−1^ at 396 W kg^−1^)^28^, activated carbon//Ni(OH)_2_/3D Ni (21.8 Wh kg^−1^ at 660 W kg^−1^)^18^, NiCo_2_S_4_//carbon (22.8 Wh kg^−1^ at 160 W kg^−1^)^29^, MnO_2_/graphene//porous carbon (46.7 Wh kg^−1^ at 100 W kg^−1^)^17^, Co_3_O_4_//activated carbon (16.42 Wh kg^−1^ at 200 W kg^−1^)^30^, graphene/porous carbon//GSP-LDH (41.2 Wh kg^−1^ at 185 W kg^−1^)^31^, ZnCo_2_O_4_/MnO_2_//a-Fe_2_O_3_ (37.8 Wh kg^−1^ at 648 W kg^−1^)^32^, NiO composite supercapacitors such as NiCo_2_O_4_/C//activated carbon (36 Wh kg^−1^ at 850 W kg^−1^),^33^ and NiO/N-C//N-graphene (50 Wh kg^−1^ at 740 W kg^−1^)^6^.

Non-noble or noble metal nanoparticles and their alloy materials have been widely studied for heterogeneous catalysis and electro catalysis applications^34^,^35^. However, the multishelled metal hollow spheres have been rarely reported due in part to the complex synthetic procedures. Herein, the multishelled NiO hollow spheres can be transferred into multishelled Ni hollow spheres in a hydrogen flow (350 °C for 2 h in a tube furnace) without serious morphology deformation (Supplementary Fig. S7a–f). The XRD patterns (Supplementary Fig. S7g) confirm that pure Ni hollow spheres were obtained from the NiO precursors. Hydrolytic dehydrogenation of ammonia borane (AB) for hydrogen generation was carried out to examine the catalytic performances of the catalysts^36^,^37^. The samples of 2S-Ni-HMS and 2S-Ni-HMS-CDS were selected respectively to test the H_2_ generation ability, and to compare the diffusion resistance of ions through the single and closed double Ni shells. It is clearly seen that the 2S-Ni-HMS shows a higher activity for H_2_ generation than 2S-Ni-HMS-CDS (Supplementary Fig. S7h), and approximately 75 mL H_2_ (per mole AB) was produced within 30 min, indicating complete dehydrogenation of AB (~90% purity of AB). The turnover frequency (TOFs) was calculated to be 598.5 mL H_2_ min^−1^ g^−1^_Ni_ and 328 mL H_2_ min^−1^ g^−1^_Ni_ for 2S-Ni-HMS and 2S-Ni-HMS-CDS respectively. The high activity may be attributed to the lower ion diffusion resistance of the thin single shell (2S-Ni-HMS) in comparison with the closed double shelled (2S-Ni-HMS-CDS). Thus the ion diffusion and substance exchange between the interior space and outer surroundings of the Ni hollow spheres are facilitated effectively. Here, the H_2_ generation ability of the multishelled Ni hollow spheres (598.5 mL H_2_ min^−1^ g^−1^_Ni_) at the same condition is higher than those of carbon supported Ni nanoparticles (361 mL H_2_ min^−1^ g^−1^_Ni_)^38^, nanoporous Ni particles (532 mL H_2_ min^−1^ g^−1^_Ni_)^39^, and the single-shelled Ni hollow microspheres (592 mL H_2_ min^−1^ g^−1^_Ni_)^40^. Therefore, the multishelled Ni hollow spheres should be practicable catalyst for H_2_ generation and various catalytic reactions due to its high performance, magnetically recycling property, low cost and easy preparation.

## Conclusion

In summary, two series of multishelled NiO hollow microspheres with an outer thin single shell and exterior closed double-shells were facilely prepared by a resin template sacrificial method. The number of shells, the shell spacing and exterior shell structure can be easily controlled by adjusting the synthetic parameters such as Ni^2+^ concentration and annealing rate. Due to their high specific surface area, the low internal resistances and Warburg impedance, 3S-, 2S- and 1S-NiO-HMS with a large shell spacing and exterior thin single shell shows high capacitance performances. The capacitance capacities of 1236 and 1057 F g^−1^ were obtained at 2 A g^−1^ for the 3S-, 2S-NiO-HMS respectively. A 3S-NiO-HMS//RGO@Fe_3_O_4_ asymmetric capacitor shows an ultrahigh energy density of 51.0 Wh kg^−1^ at a power density of 800 W kg^−1^. Even at a high power density of 8,000 W kg^−1^, the capacitor still delivers an energy density of 22.7 Wh kg^−1^. The excellent performance is superior to that of the previous NiO-based supercapacitors and Ni, Co and Mn-based oxide hollow spheres. Remarkably, the asymmetric supercapacitor manifests very high cycling stability and still delivers 78.8% of its initial capacitance even after 10,000 cycles. In addition, multishelled Ni hollow spheres were easily obtained from the corresponding multishelled NiO hollow spheres, and they showed a high H_2_ generation ability of 598.5 mL H_2_ min^−1^ g^−1^_Ni_ for 2S-NiO-HMS. Therefore, the multishelled Ni hollow spheres offer a promising future for H_2_ generation, due to their high performance, magnetically recycling property, low cost and easy preparation.

## Methods

### Chemicals

Tri-block copolymer Pluronic F127 (EO_106_PO_70_EO_106_, EO = ethylene oxide, PO = propylene oxide, 99.0 wt%) and cetyltrimethyl ammonium bromide (CTAB) were purchased from Sigma-Aldrich. Ethylene glycol (AR) was obtained from Sinopharm Chemical Reagent Co., Ltd. 2,4-Dihydroxybenzoic acid (98%) was bought from Aladdin. Formaldehyde, NH_3_∙H_2_O and nickel nitrate were purchased from Tianjin Damao Chemical Reagent Co., Ltd (China). Nickel foam was received from Lifeixin Co., Ltd. (China). Carbon black was purchased from Xinxing and polytetrafluoroethylene (PTFE) emulsion from Daikin Co., Ltd. (China).

### Synthesis of RF (resin) microspheres

The RF microspheres were synthesized by using 2,4-dihydroxybenzoic acid and formaldehyde as precursors. Firstly, 0.5 g of F127 and 0.2 g of CTAB were added into 40 mL of deionized water and 16 mL of ethanol. The above solution was stirred at 30 °C for 15 min, and then 0.2 mL of NH_3_·H_2_O was injected with further stirring at 30 °C for 50 min. After that, 0.56 g of 2,4-dihydroxybenzoic acid was added, which was stirred at 30 °C for additional 30 min, and then 0.56 mL of formaldehyde was injected. The above mixture was stirred at 30 °C for 24 h. At last, the mixture was heated at 100 °C for 24 h in the Teflon-lined auto-clave in the oven. The products were washed with water and ethanol several times and then dried at 60 °C in air overnight to obtain RF microspheres.

### Synthesis of multishelled NiO hollow microspheres

In a typical synthesis of multishelled NiO hollow microspheres, 200 mg of RF microspheres were dispersed homogeneously in 80 mL of ethylene glycol under ultrasonication condition. And then 100 mL of nickel nitrate solution (ethylene glycol) was added into the above dispersion, which was stirred at 80 °C for 7 h. The products were washed with ethanol for several times and dried in the oven overnight. After that, the multishelled NiO hollow microspheres were obtained by calcining the above microspheres in a muffle furnace from 50 °C to 550 °C, and held at 550 °C for 4 h under air condition. The numbers of shell and the distance between adjacent shells were controlled by the absorbing time (0.5 and 7 h), concentration (0.05, 0.1, 0.5 and 1 M) of nickel nitrate and heating ramp (0.5, 2, 5, and 10 °C·min^−1^).

### Synthesis of multishelled Ni hollow microspheres

The multishelled NiO hollow microspheres were reduced by hydrogen (60 mL min^−1^) at 350 °C for 2 h in the tube furnace to obtain multishelled Ni hollow microspheres. The tube furnace should be swept by hydrogen for 20 min to avoid danger previously.

### Electrochemical performance tests

A three-electrode system was used to measure the electrochemical characteristics of the multishelled NiO hollow microspheres. The NiO microspheres were used as the working electrode, a standard calomel electrode (SCE) as the reference electrode, 2 M KOH aqueous solution as the electrolyte and a graphite rod as the counter electrode. The multishelled NiO hollow microspheres, carbon black and 5 wt% PTFE (80 wt%: 10 wt%: 10 wt%) were mixed to form a paste and then pressed onto the precleaned nickel foam (~0.5 mg/cm^2^), which was dried at 60 °C in the oven for 12 h. The electrochemical performance was characterized by cyclic voltammetry (CV) and galvanostatic charge/discharge test between −0.05 and 0.45 V vs. SCE on an IviumStat Electrochemical Workstation (Netherlands). Electrochemical impedance spectroscopy (EIS) measurements were also made under an open circuit potential with AC turbulence voltage amplitude of 5 mV in the frequency range of 100 kHz–0.01 Hz. The specific capacitance was calculated from the following equation, *C* = *I*Δ*t*/*m*Δ*V*. Where I (A) is the discharge current, Δt (s) is the discharge time, m (g) is the mass of the electroactive materials and ΔV (V) is the discharge voltage range.

Asymmetric capacitor: A LAND-CT2001A battery testing system (Wuhan, China) was used to measure the electrochemical performance of multishelled NiO hollow microspheres//RGO@Fe_3_O_4_ asymmetric capacitor. The RGO@Fe_3_O_4_ composites, carbon black and 5 wt% PTFE (80 wt%: 10 wt%: 10 wt%) were finely mixed in an agate mortar to form a paste and then pressed onto the precleaned nickel foam, which was dried at 60 °C in the oven for 12 h. Based on the specific capacitance of each electrode and the principle of charge balance between two electrodes, q^+^ = q^−^ should be satisfied, where q^+^ is the positive charges and q^−^ is the negative charges stored by electrode (q = C × ΔE × m, where C (F g^−1^) is the specific capacitance of electrode, ΔE (V) is the potential window during discharge process and m (g) is the mass of electrode). The mass ratio of multishelled NiO to the RGO@Fe_3_O_4_ was determined to be ~0.3. The electrochemical properties of the asymmetric full-cell device were investigated under a two-electrode cell configuration with multishelled NiO as a positive electrode, RGO@Fe_3_O_4_ composites as a negative electrode and porous polypropylene membrane as a separator in 2.0 M KOH electrolyte solution. The specific capacitance (*C* = *I*Δ*t*/*m*Δ*V*, F g^−1^), energy density (E = *C*Δ*V*^2^/2, Wh kg^−1^) and power density (P = *E*/Δ*t*, W kg^−1^) were calculated respectively. Where I (A) is the discharge current, Δt (s) is the discharge time, m (g) is the total mass of samples for both electrodes and ΔV (V) is the discharge voltage.

### Synthesis of RGO@Fe_3_O_4_ composites

Graphene oxide was obtained from Hengqiu Tech. Inc., China. A facile solvothermal method was used to prepare RGO@Fe_3_O_4_ composites^26^. Typically, 70 mL of GO aqueous solution was heated to 70 °C under magnetic stirring, to which 15 mL of FeCl_3_·6H_2_O (0.2 g) and FeCl_2_·4H_2_O (0.08 g) aqueous solution was added. The above mixture was stirred at 70 °C under nitrogen atmosphere for 15 h. After that, 8 mL of NH_3_·H_2_O was injected into the mixture. At last, the mixture was heated at 150 °C for 2 h in the Teflon-lined auto-clave in the oven. The products were washed with water and ethanol several times and then dried at 60 °C to obtain RGO@Fe_3_O_4_ composites. The electrochemical measurements of RGO@Fe_3_O_4_ nanocomposites were also conducted in a 2 M KOH solution (Supplementary Fig. S6).

### Hydrolytic dehydrogenation of ammonia borane (AB)

10 mL of well dispersed multishelled Ni microspheres (10 mg) dispersion was placed into a three necked round-bottom flask. And then 10 mL of ammonia borane (35 mg) was added into the above flask. The volume of released H_2_ was monitored by an inverted and water filled gas burette. The catalytic hydrolysis of AB can produce 3 mol of H_2_ per mol of AB in theory 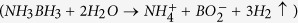.

### Characterization

The morphologies of the multishelled NiO hollow microspheres were monitored using a field emission scanning electron microscopy (FE-SEM, Nova NanoSEM 450). The microstructures and energy-dispersive X-ray spectroscopy elemental mapping images were obtained by a JEOL-2010F transmission electron microscopy (TEM). X-ray diffraction (XRD) patterns were obtained with a D/MAX-2400 diffractometer (Cu Kα radiation, 0.154 nm). The nitrogen adsorption-desorption isotherms were measured at 77.35 K using a Micromeritics AUTOSORB-1-MP.

## Additional Information

**How to cite this article**: Qi, X. *et al*. Multishelled NiO Hollow Microspheres for High-performance Supercapacitors with Ultrahigh Energy Density and Robust Cycle Life. *Sci. Rep.* **6**, 33241; doi: 10.1038/srep33241 (2016).

## Supplementary Material

Supplementary Information
