# Supplementary material for: Multishelled NiO Hollow Microspheres for High-performance Supercapacitors with Ultrahigh Energy Density and Robust Cycle Life
Source: Sci Rep. 2016 Sep 12;6:33241. doi: 10.1038/srep33241 (PMC5018958; doi:10.1038/srep33241)
Supplement: Supplementary Information [file srep33241-s1.pdf]

Supporting Information

Multishelled NiO Hollow Microspheres for High-performance Supercapacitors with Ultrahigh Energy Density and Robust Cycle Life

Xinhong Qi, Wenji Zheng, Xiangcun Li, Gaohong He*

State Key Laboratory of Fine Chemicals, Chemical Engineering Department, Dalian University of Technology

Linggong Road 2#, Dalian 116024, China

E-mail: lixiangcun@dlut.edu.cn

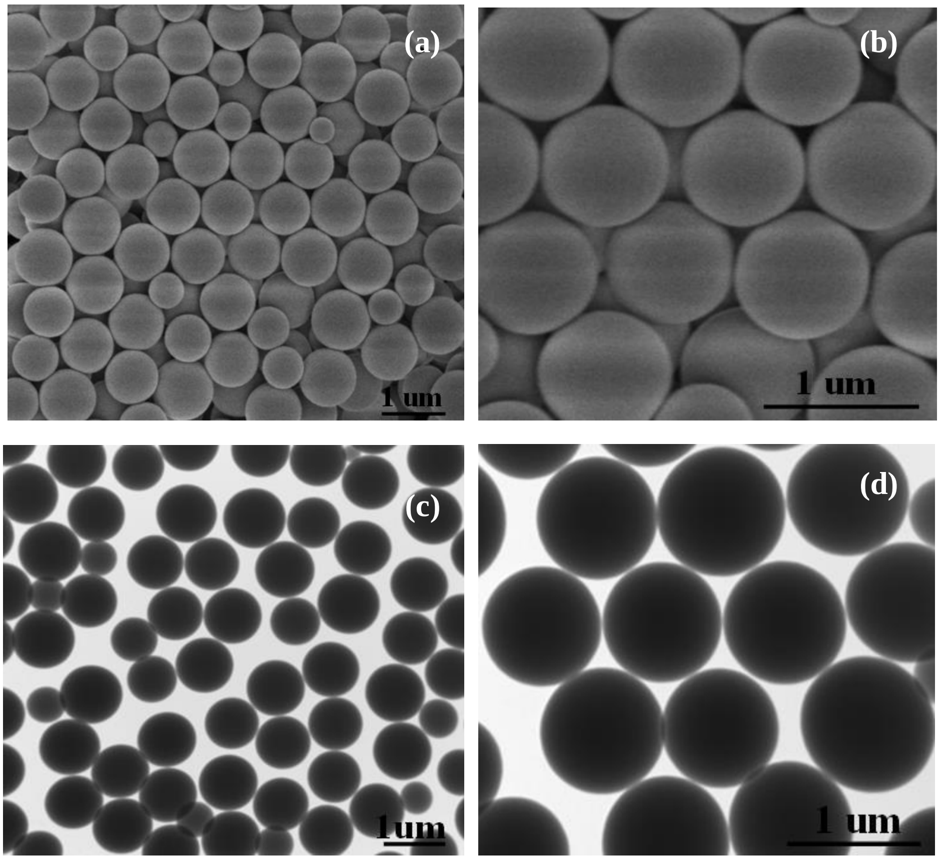

Figure S1 (a, b) SEM and (c, d) TEM images of the resin microspheres prepared by a mild hydrothermal method for preparation of multishelled NiO hollow spheres

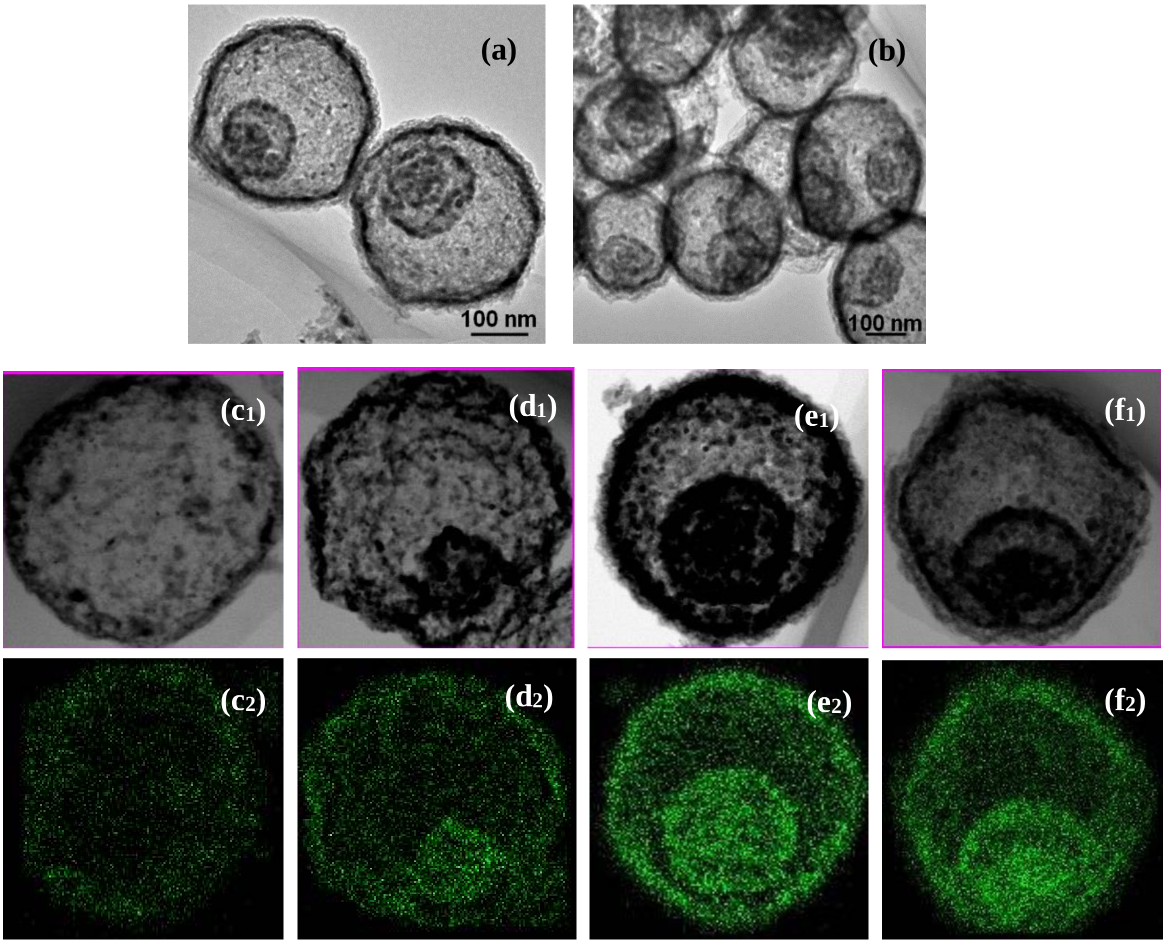

Figure S2 (a) Quadruple-shelled NiO hollow microspheres with closed exterior double-shells obtained at an annealing rate of 10 ℃/min (soaking 7 h in 0.5 M Ni^{2+} solution), (b) quadruple-shelled NiO hollow microspheres upon soaking RF templates in 1.0 M Ni^{2+} solution for 7 h (2 ℃/min), (c_1-f_1) and (c_2-f_2) shows scanning transmission electron microscope (STEM) images and energy dispersive X-ray spectroscopy (EDS) elemental mapping of 1S-, 2S, 3S-NiO-HMS and 4S-NiO-HMS-CDS.

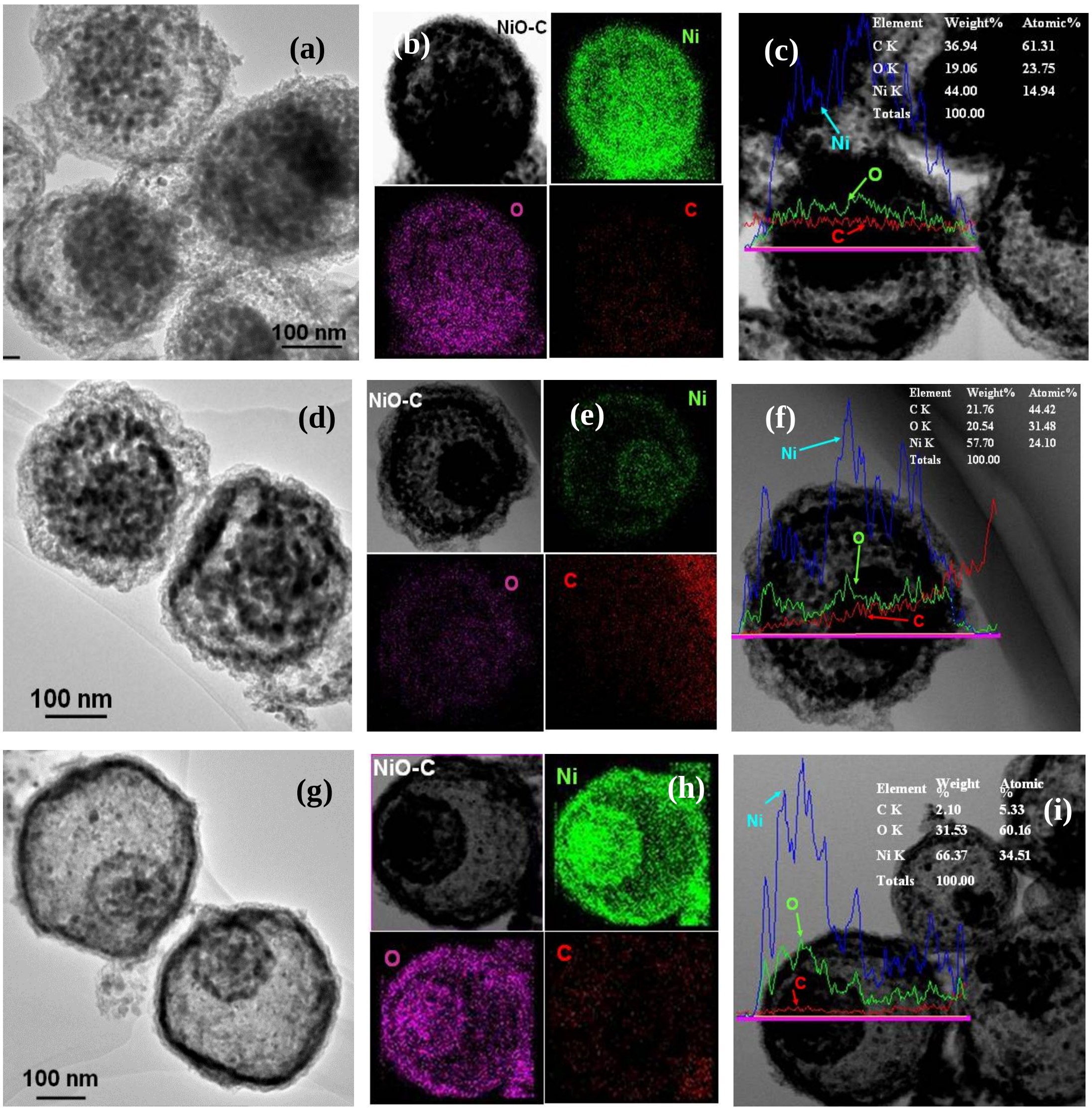

Figure S3 Formation mechanism of the novel metal oxide nanostructures, investigations using TEM, STEM, EDS elemental mapping and line scanning to monitor the morphological evolution and formation process as a function of the annealing time, (a-c) 0.5 h, (d-f) 1.5 h, (g-i) 3 h.

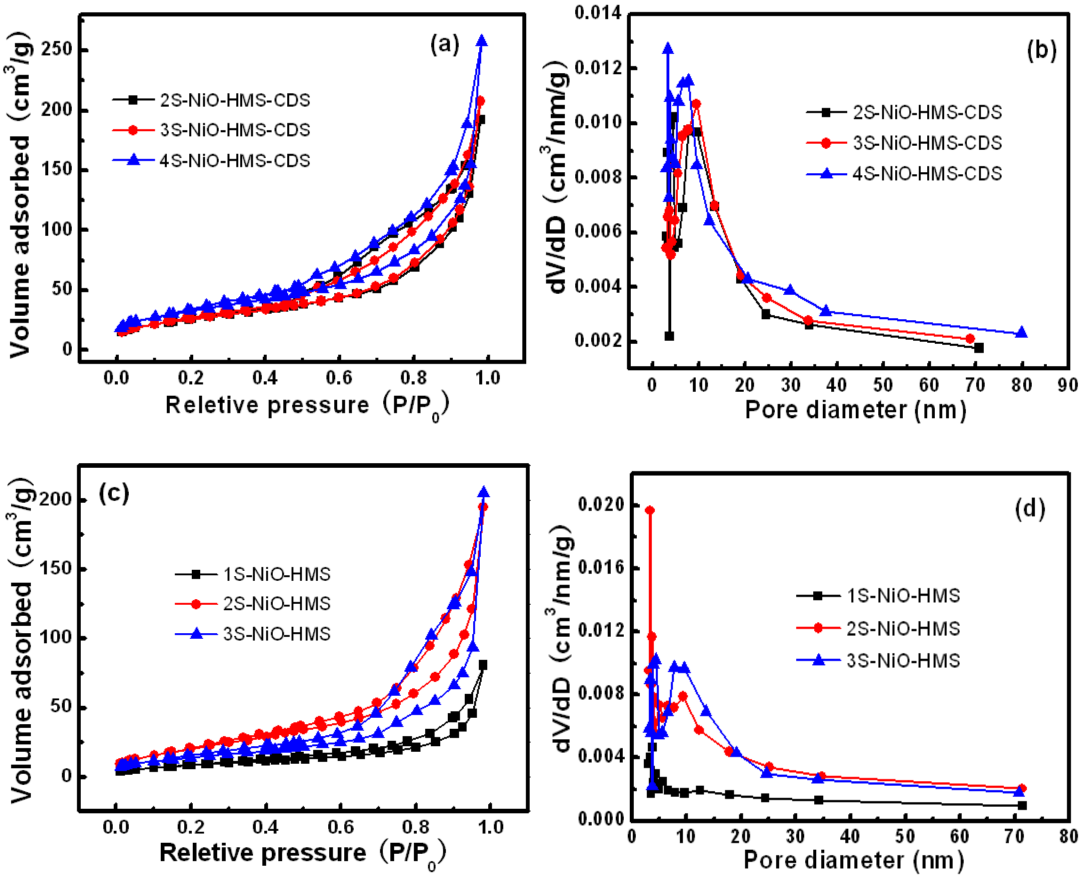

Figure S4 Nitrogen adsorption-desorption isotherms and the corresponding BJH pore size distribution curves calculated from the desorption branch.

Table S1 Surface area and porosity characteristics of multishelled NiO hollow microspheres

Samples	BET surface area (m^2/g)	Pore volume (cm^3/g)	Average pore diameter (nm)
3S-NiO-HMS	93.28	0.334	3.760
2S-NiO-HMS	88.18	0.300	3.733
1S-NiO-HMS	32.73	0.122	3.325
2S-NiO-HMS-CDS	95.60	0.314	4.338
3S-NiO-HMS-CDS	94.15	0.327	3.584
4S-NiO-HMS-CDS	117.8	0.393	3.323

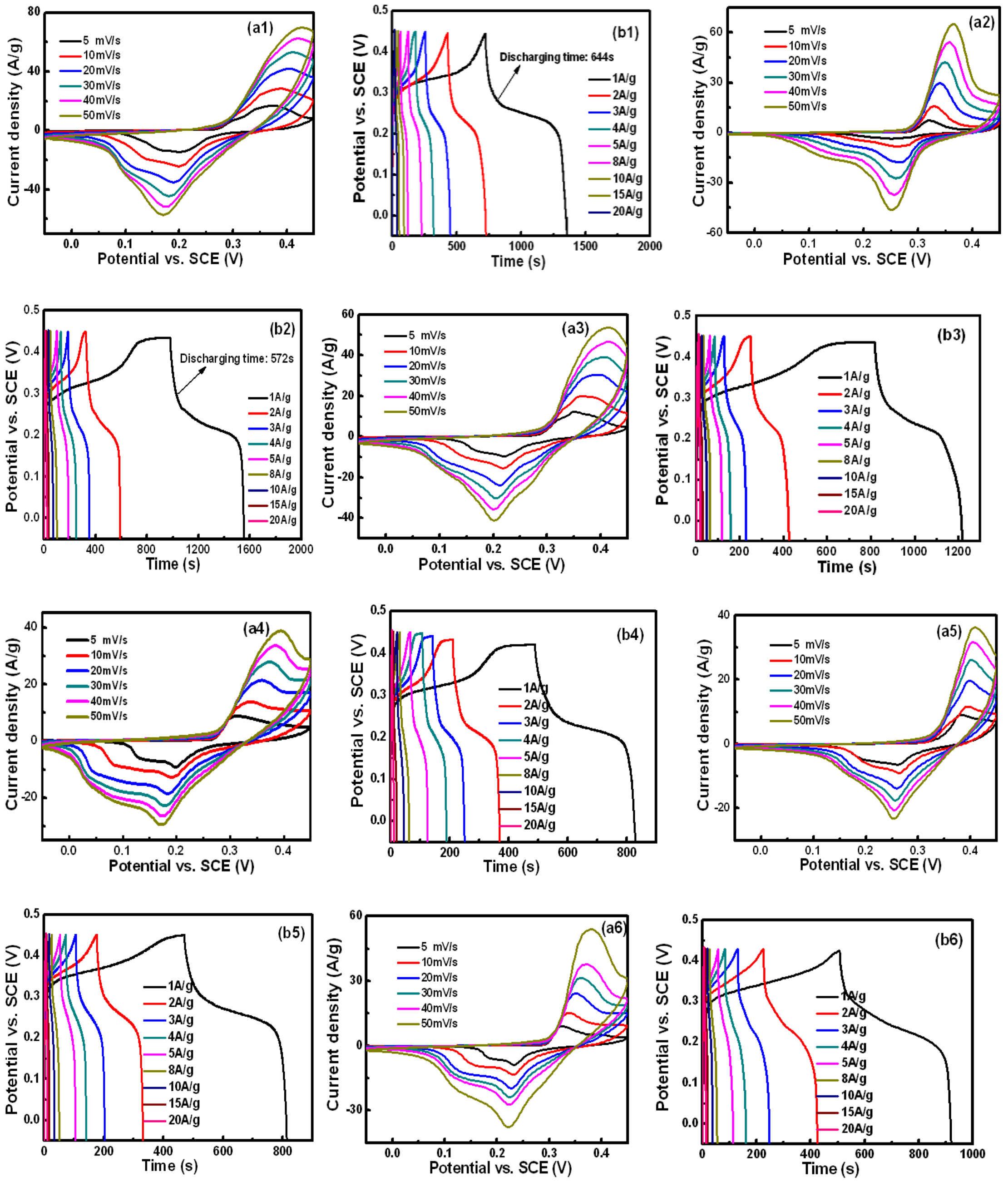

Figure S5 (a1-a6) CV curves of NiO microspheres at different scan rates; (b1-b6) charge/discharge curves of NiO microspheres at different current densities, (a1, b1- a6, b6) represent 3S-NiO-HMS, 2S-NiO-HMS, 1S-NiO-HMS, 2S-NiO-HMS-CDS, 3S-NiO-HMS-CDS and 4S-NiO-HMS-CDS respectively.

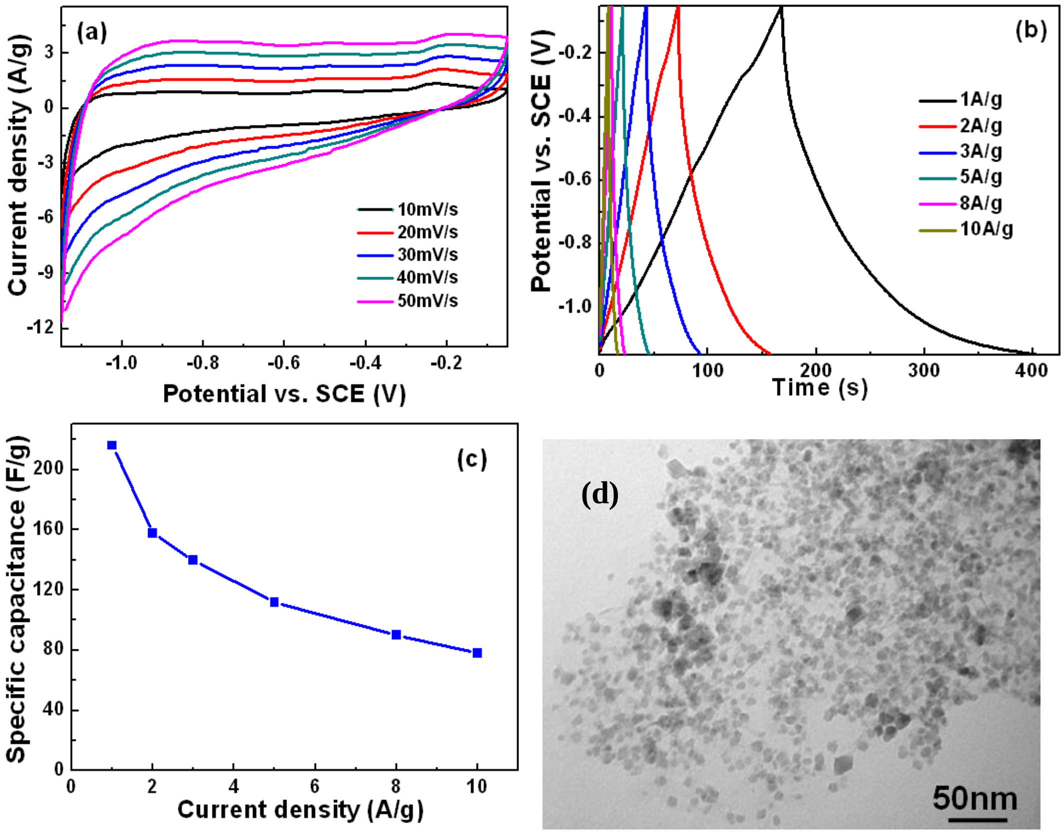

Figure S6 (a) CV curves of the RGO@Fe_3O_4 composite electrode at different scan rates, (b) galvanostatic charge/discharge curves and (c) specific capacitance of the RGO@Fe_3O_4 electrode at different current densities, (d) TEM image of the RGO@Fe_3O_4 composite electrode materials.

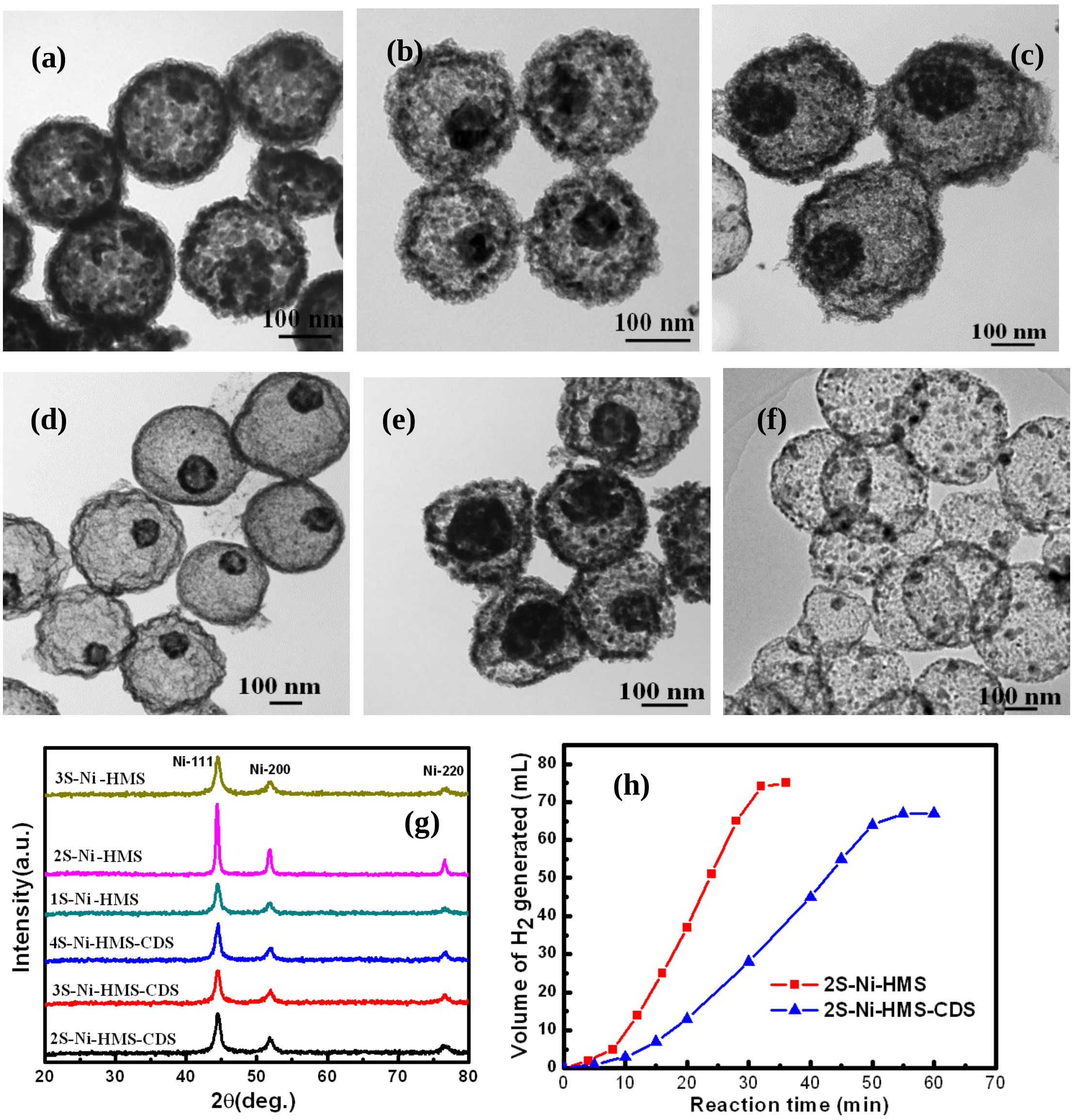

Figure S7 TEM images: (a), (b) and (c) the corresponding double-, triple-, and quadruple-shelled hollow Ni microspheres with closed exterior double-shells (2S-Ni-HMS-CDS, 3S-Ni-HMS-CDS, 4S-Ni-HMS-CDS); (d), (e) and (f) double-, triple-, and single-shelled hollow Ni microspheres; (2S-Ni-HMS, 3S-Ni-HMS, 1S-Ni-HMS); (g) XRD patterns of various Ni hollow spheres; (h) hydrogen generation with 2S-Ni-HMS and 2S-Ni-HMS-CDS as catalysts respectively.

Table S2 Comparison of the maximum energy densities, corresponding average power densities based on active materials and voltage range of some reported nickel or cobalt oxide based asymmetric supercapacitors, other typical asymmetric supercapacitors and the present work

Positive materials//negative materials	Energy density ($W\,h \cdot kg^{-1}$)	Power density ($W \cdot kg^{-1}$)	Voltage range (V)	Ref.
CNT/NiO//porous carbon polyhedrons	23.4	1000	0-1.6	[1]
NiO//carbon	11.3	920	0.8-1.8	[2]
NiO-Ni//activated carbon	19.1	1100	0-1.5	[3]
$Ni(OH)_2$/graphene//graphene	30.0	800	0-1.6	[4]
$NiCo_2O_4/Co_{0.33}Ni_{0.67}(OH)_2$//CMK-3-ASC	31.2	396	0-1.6	[5]
$Ni(OH)_2$/3D Ni //activated carbon	21.8	660	0-1.3	[6]
$NiCo_2S_4$//carbon	22.8	160	0-1.5	[7]
MnO_2/graphene//porous carbon	46.7	100	0-2	[8]
Co_3O_4 hollow spheres//activated carbon	16.42	200	0-1.6	[9]
Co-Al hydroxide nanosheets graphene//carbon	41.2	185.4	0-1.6	[10]
$ZnCo_2O_4/MnO_2$nanotube arrays //a-Fe_2O_3	37.8	648	0-1.3	[11]
$NiCo_2O_4$/C hollow spheres//activated carbon	36	852	0-1.6	[12]
NiO/N-C hollow spheres//N-graphene	50	740	0-1.5	[13]
$Ni_xCo_{3-x}O_4$ nanowire//activated carbon	37.4	163	0-1.6	[14]
NiCo2O4 nanosheets // activated carbon	15.42	500	0-1.5	[15]
$CoNi_3O_4$/Carbon//AC	29.1	130.4	0-1.8	[16]
Co3O4@Ni(OH)2//RGO	31.5	100	0-1.5	[17]
$Co_{0.5}Ni_{0.5}(OH)_2$/graphene/CNT//AC/FWNT	41	210	0-1.4	[18]
$NiCo_2S_4$ hollow spheres//graphene/carbon spheres	42.3	476	0-1.6	[19]
H–CoOx@$Ni(OH)_2$//RGO@Fe_3O_4	45.3	1010	0-1.6	[20]
**3S-NiO-HMS//RGO@Fe_3O_4**	**51.0**	**800**	**0-1.6**	**This work**

References

1 Yi, H., Wang, H., Jing, Y., Peng, T. & Wang, X. Asymmetric supercapacitors based on carbon nanotubes@ NiO ultrathin nanosheets core-shell composites and MOF-derived porous carbon polyhedrons with super-long cycle life. *J. Power Sources* **285**, 281-290 (2015).

2 Lu, X. *et al.* High Energy Density Asymmetric Quasi-Solid-State Supercapacitor Based on Porous Vanadium Nitride Nanowire Anode. *Nano Lett.* **13**, 2628-2633 (2013).

3 Inoue, H., Namba, Y. & Higuchi, E. Preparation and characterization of Ni-based positive electrodes for use in aqueous electrochemical capacitors. *J. Power Sources* **195**, 6239-6244 (2010).

4 Yan, J. *et al.* Advanced Asymmetric Supercapacitors Based on $Ni(OH)_2$/Graphene and Porous Graphene Electrodes with High Energy Density. *Adv. Funct. Mater.* **22**, 2632-2641 (2012).

5 Xu, K. *et al.* Design and synthesis of 3D interconnected mesoporous $NiCo_2O_4$@$Co_xNi_{1-x}(OH)_2$ core–shell nanosheet arrays with large areal capacitance and high rate performance for supercapacitors. *J. Mater. Chem. A* **2**, 10090 (2014).

6 Su, Y.-Z., Xiao, K., Li, N., Liu, Z.-Q. & Qiao, S.-Z. Amorphous $Ni(OH)_2$ @ three-dimensional Ni core-shell nanostructures for high capacitance pseudocapacitors and asymmetric supercapacitors. *J. Mater. Chem. A* **2**, 13845-13853 (2014).

7 Kong, W., Lu, C., Zhang, W., Pub, J. & Wang, Z. Homogeneous core-shell $NiCo_2S_4$ nanostructures supported on nickel foam for supercapacitors. *J. Mater. Chem. A* **3**, 12452-12460 (2015).

8 Zhao, Y. *et al.* High-performance asymmetric supercapacitors based on multilayer MnO_2 /graphene oxide nanoflakes and hierarchical porous carbon with enhanced cycling stability. *Small* **11**, 1310-1319 (2015).

9 Wang, Y. *et al.* Synthesis of 3D-nanonet hollow structured Co_3O_4 for high capacity supercapacitor. *ACS Appl. Mater. Inter.* **6**, 6739-6747 (2014).

10 Wu, X., Jiang, L., Long, C., Wei, T. & Fan, Z. Dual Support System Ensuring Porous Co-Al Hydroxide Nanosheets with Ultrahigh Rate Performance and High Energy Density for Supercapacitors. *Adv. Funct. Mater.* **25**, 1648-1655 (2015).

11 Ma, W., Nan, H., Gu, Z., Geng, B. & Zhang, X. Superior performance asymmetric supercapacitors based on $ZnCo_2O_4$@MnO_2 core-shell electrode. *J. Mater. Chem. A* **3**, 5442-5448 (2015).

12 Lei, Y., Wang, Y., Yang, W., Yuan, H. & Xiao, D. Self-assembled hollow urchin-like $NiCo_2O_4$ microspheres for aqueous asymmetric supercapacitors. *RSC Adv.* **5**, 7575-7583 (2015).

13 Kim, S. Y. *et al.* Nickel oxide encapsulated nitrogen-rich carbon hollow spheres with multiporosity for high-performance pseudocapacitors having extremely robust cycle life. *Energ. Environ. Sci.* **8**, 188-194 (2015).

14 Wang, X., Yan, C., Sumboja, A. & Lee, P. S. High performance porous nickel cobalt oxide nanowires for asymmetric supercapacitor. *Nano Energy* **3**, 119-126 (2014).

15 Lu, X.-F. *et al.* Hierarchical $NiCo_2O_4$ nanosheets@hollow microrod arrays for high-performance asymmetric supercapacitors. *J. Mater. Chem. A* **2**, 4706-4713 (2014).

16 Zhu, J. *et al.* 3D Carbon/Cobalt-Nickel Mixed-Oxide Hybrid Nanostructured Arrays for Asymmetric Supercapacitors. *Small* **10**, 2937-2945 (2014).

17 Tang, C.-h., Yin, X. & Gong, H. Superior Performance Asymmetric Supercapacitors Based on a Directly Grown Commercial Mass 3D Co_3O_4@$Ni(OH)_2$ Core-Shell Electrode. *ACS Appl.*

Mater. Inter. **5**, 10574-10582 (2013).

18 Cheng, Y., Zhang, H., Varanasi, C. V. & Liu, J. Improving the performance of cobalt-nickel hydroxide based self-supporting electrodes for supercapacitors using accumulative approaches. *Energ. Environ. Sci.* **6**, 3314-3321 (2013).

19 Shen, L. *et al.* Formation of nickel cobalt sulfide ball-in-ball hollow spheres with enhanced electrochemical pseudocapacitive properties. *Nat. Commun.* **6** (2015).

20 Zhu, J. *et al.* Hydrogenated CoO_x nanowire@$Ni(OH)_2$ nanosheet core-shell nanostructures for high-performance asymmetric supercapacitors. *Nanoscale* **6**, 6772-6781 (2014).
